# TRENDS IN DIGITAL DEVICE ACCESS AMONG HISPANIC OLDER ADULTS IN THE US (2011–2021)

**DOI:** 10.1093/geroni/igae098.2570

**Published:** 2024-12-31

**Authors:** Xiayu Chen, Yanjun Dong, Karla Sanabria Véaz, Danan Gu, Kun Wang

**Affiliations:** University of Illinois Urbana-Champaign, Champaign, Illinois, United States; SUNY Albany, Albany, New York, United States; University of Illinois Urbana-Champaign, Champaign, Illinois, United States; NA, Suzhou, Jiangsu, China (People’s Republic); Binghamton University, Binghamton, New York, United States

## Abstract

Despite the general increase in digital technology use among U.S. older adults within the past decade, Hispanic older adults may still face a notable digital gap due to socioeconomic disparities. Guided by the intersectionality lens, the study aims to examine (1) the trends of digital device access between Hispanic and non-Hispanic White older adults in the U.S. from 2011 to 2021 and (2) the effect of gender, education and income factors on the digital access disparity. We used 11 waves of National Health and Aging Trend data, and identified 38,253 observations from 8,503 community-dwelling older adults aged 70 and above for the analysis. Applying weighted multilevel logistic regressions, we found that compared to non-Hispanic White older adults, those who were Hispanic had significantly lower odds of having digital device access (OR = 0.42, 95% CI = 0.25 - 0.70). However, the gap in digital device access has narrowed over time. Meanwhile, specific factor-stratified models revealed that the digital access between Hispanic and non-Hispanic White older adults was significantly reduced with time among both men and women, those with low education, and those with low income, but not among those with higher education and medium-to-high income. Our findings highlight notable progress in narrowing the digital access gap for Hispanic older adults in 2011 to 2021. Nevertheless, disparities persist, necessitating continued efforts in tailored policies and community programs to address the complex factors contributing to digital inequalities.

